# Rapid N-Terminal Pro-B-Type Natriuretic Peptide (NT-proBNP) Kit as a Differentiating Tool for Acute Dyspnea in a Resource-Limited Setting

**DOI:** 10.7759/cureus.48306

**Published:** 2023-11-05

**Authors:** Sweta Sahu, Devarsh N Shah, Roopeessh Vempati, Pallavi Roy Kandhi, Mihirkumar P Parmar, Sanjay Bethanabotla, Shardool Gadgil, Prerna Chandra, Sangamesh N Malipatil, Yash Patel, Balaganesh Natarajan, Thrilok Chander Bingi

**Affiliations:** 1 Surgery, JJM (Jagadguru Jayadeva Murugarajendra) Medical College, Davanagere, IND; 2 Medicine and Surgery, Medical College Baroda, Vadodara, IND; 3 Internal Medicine, Gandhi Medical College, Secunderabad, IND; 4 Cardiology, Heart & Vascular Institute, Detroit, USA; 5 Internal Medicine, Kakatiya Medical College, Warangal, IND; 6 Internal Medicine, Gujarat Medical Education and Research Society, Vadnagar, IND; 7 Internal Medicine, Osmania Medical College, Hyderabad, IND; 8 Medicine and Surgery, Lokmanya Tilak Municipal General Hospital and Medical College, Mumbai, IND; 9 Internal Medicine, Deccan College of Medical Sciences, Hyderabad, IND; 10 Medicine, Mahadevappa Rampure Medical College, Kalaburagi, IND; 11 Neurology, St. George's University School of Medicine, Saint George's, GRD; 12 General Medicine, Gandhi Hospital, Secunderabad, IND

**Keywords:** cardiopulmonary diseases, resource limited setting, nt-probnp, severe dyspnea, acute heart faiure

## Abstract

Introduction

Dyspnea is among the most prevalent symptoms experienced by patients presenting as an emergency. The underlying etiology is often a cardiovascular or pulmonary condition, of which heart failure is recognized as a major contributor. The differentials are primarily established based on the patient's clinical presentation and physical examinations but are not conclusive. Of the various investigations undertaken to determine the cause of dyspnea, the biomarker N-Terminal Pro-B-Type Natriuretic Peptide (NT-proBNP) was found to be significantly associated with heart failure. Its level has been proven to be in direct correlation with the severity of the disease. This study demonstrates the usability of an economical rapid test kit in measuring NT-ProBNP levels to help differentiate the cause of dyspnea in the presenting patient in a resource-limited setting.

Methodology

We studied 115 participants from a tertiary care center in India, which included 70 males and 45 females aged ≤30 to ≥75 years, presenting with shortness of breath. Rapid NT-ProBNP tests were conducted alongside recording their symptoms, vitals, examination findings, and other parameters. They were also classified according to New York Heart Association (NYHA) Classification, and further investigated.

Results

The study elucidated the efficacy and accuracy of the rapid kits in determining NT-ProBNP levels, and its relation with the severity and prognosis of heart failure. The kits utilized had a sensitivity of greater than 93% for ruling out heart failure as a cause of dyspnea, and a sensitivity of greater than 96% for ruling out elevated NT-ProBNP levels in general. Other parameters such as presenting symptoms and vitals were also analyzed, establishing a correlation with NT-ProBNP levels.

Conclusion

This study guided us in understanding the effective utilization of the rapid testing kits for emergency care, minimizing the burden on other limited resources. The lower cost and ease of use would serve as a quick means of reaching a conclusive diagnosis, especially in an emergency, which in turn would aid in receiving timely and specific treatment. These kits could act as a stepping stone in creating a sustainable and efficient healthcare system for patients as well as healthcare workers.

## Introduction

Dyspnea is a common reason for emergency department admissions worldwide. Diagnosis is often difficult in patients presenting with breathlessness as there are multiple causes for the same. Usually, this condition is diagnosed clinically due to the limited availability of confirmatory diagnostic tools and, therefore, the use of biomarkers at the point of care might help in narrowing the diagnosis [1,2]. Heart failure, a major cause of dyspnea resulting from cardiovascular disease, is also a leading global cause of death. It has complex pathophysiological processes, and it is crucial to detect it in the early stages for better hospitalization, disease management, and overall better prognosis. Heart failure patients have a chronic course of disease but often present acutely in the emergency department as sudden onset dyspnea [3]. A study conducted by the European Society of Cardiology reports that within a one-year period, the combined endpoint of mortality or heart failure hospitalization had a rate of 36% for acute heart failure (AHF) and 14.5% for chronic heart failure (CHF). This data highlights a significantly higher risk of adverse outcomes in patients with AHF compared to CHF within the specified timeframe. Understanding these differences can aid in tailoring appropriate management strategies and interventions to improve patient outcomes and reduce the burden of heart failure [4].

We commonly diagnose heart failure using the Framingham criteria, requiring two major criteria or one major and two minor criteria for reaching the final diagnosis [3]. Major criteria include acute pulmonary edema, cardiomegaly, hepatojugular reflux, neck vein distention, paroxysmal nocturnal dyspnea or orthopnea, pulmonary rales, third heart sound (s3 gallop), weight loss of 4.5 kg or more in five days in response to treatment, central venous pressure greater than 16 cm of water, and radiographic cardiomegaly. Minor criteria include ankle edema, dyspnea on exertion, hepatomegaly, nocturnal cough, pleural effusion, tachycardia (heart rate greater than 120 beats per minute), and a decrease in vital capacity by one-third of the maximal value recorded. Patients can also be classified according to the New York Heart Association (NYHA) classification based on the symptoms. NYHA Class-1 includes symptom onset with more than an ordinary level of activity, Class-2 includes symptom onset with an ordinary level of activity, Class-3 includes symptom onset with minimal activity (wherein Class-3a is used when there is no dyspnea at rest, and Class-3b is used when there is recent onset dyspnea at rest), and Class-4 includes symptoms at rest [3]. Two-dimensional (2D) echocardiography is generally preferred for the evaluation of heart failure as it aids in determining the ejection fraction and dimensions of the heart chambers, but due to high cost and no routine availability in the emergency department, its use is very limited. Hence, a shift of focus toward recent diagnostic markers like N-Terminal Pro-B-Type Natriuretic Peptide (NT-proBNP) levels is advisable for prompt diagnosis and management of heart failure [5].

Atrial natriuretic peptide (ANP) and brain natriuretic peptide (BNP) are two peptides secreted by the heart primarily in response to stretching of the heart muscle caused by increased volume load. These peptides are initially synthesized as preprohormones and later converted into active endocrine peptides (ANP, BNP) and their N-terminal prohormone fragments, detectable in the bloodstream. The natriuretic peptide system is most prominently activated in cases of ventricular dysfunction [6]. However, in patients with edematous disorders, where there is an increase in atrial tension or central blood volume, such as renal failure or ascitic liver cirrhosis, natriuretic peptides increase can be observed. Studies have demonstrated that among all the tested neurohormones, cardiac natriuretic peptides were the most effective markers for identifying heart failure in chronic heart failure patients and during the subacute phase of myocardial infarction. Moreover, they were found to be highly predictive of both morbidity and mortality in these patients [6,7].

Though there are recent advances in the use of biomarkers such as BNP and NT-ProBNP, the use of it in tertiary hospitals is limited. NT-ProBNP assays are traditionally performed using laboratory-based whole blood immunoassay, but lately, rapid diagnostic kits have been developed that can measure serum BNP levels in the emergency department itself within 10-15 minutes. These kits have proved to be extremely affordable and equally efficient as compared to traditional assays [8,9]. This study will help us show how the rapid NT-ProBNP test can be effective in early differential diagnosis of acute dyspnea in an emergency. This rapid test potentially aids in early diagnosis and, therefore, subsequent timely management of congestive heart failure.

## Materials and methods

We analyzed 115 adult patients presenting with dyspnea in the emergency department of Gandhi Medical College, Secunderabad, India, from January 2023 to April 2023. Sample size calculation was done using the following Cochrane formula, based on which, the required sample size was estimated to be 113.

$\eta = (Z^2pq)/e^2$

where η: sample size; Z: 1.96 (value of standard distribution for a confidence interval of 95%); p: 8% (prevalence of dyspnea patients in the emergency department [2]); q: 92% (1-p); e: 5% (margin of error)

**Figure 1:**
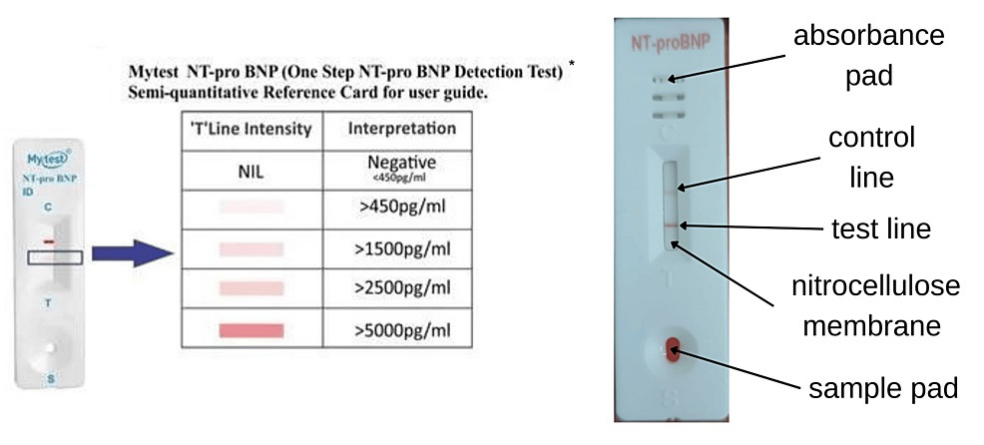
Rapid NT-proBNP kit used in the study *Mytest® NT-proBNP; Biofootprints Healthcare Pvt Ltd, New Delhi, India

Measurement of blood NT-proBNP level was performed within the first hour of admission using an NT-proBNP rapid test kit (Mytest® NT-proBNP, Biofootprints Healthcare Pvt Ltd, New Delhi, India) (Figure 1). The NT-proBNP rapid test is based on fluorescence immunoassay for the semi-quantitative determination of human NT-proBNP in whole blood, serum, or plasma, which is used as an aid in diagnosing heart failure. Approximately 2 ml venous blood was collected with consent from patients presenting with acute dyspnea. Venous blood was collected in a clot activator vacuum tube (red top) along with the blood samples routinely collected in the emergency department. Serum separated from whole blood was used for semi-quantitative rapid NT-proBNP testing. The test results were documented along with other patient details like history and vitals, examination findings, and other investigations.

The primary objective of the study was to find out the effectiveness of the rapid test using parameters such as sensitivity, specificity, positive predictive value, and negative predictive value. To estimate these, the determinants required were true positive (TP) values, false positive (FP) values, true negative (TN) values, and false negative (FN) values.

TP is positive results correctly indicating the cause of dyspnea due to the presence of heart failure, TN indicates negative results correctly indicating the cause of dyspnea is NOT due to heart failure but some other etiology, FP means positive results incorrectly indicate the cause of dyspnea to be due to heart failure when, in reality, the etiology is different, and FN indicate negative results that incorrectly indicate the cause of dyspnea to be due to etiologies other than heart failure when, in reality, the etiology is heart failure.

## Results

An observational study was conducted in the emergency medicine center at Gandhi Hospital, Secunderabad. A total of 115 adult patients with complaints of dyspnea on presentation were included in the study over a period of four months and were tested with the rapid NT-proBNP kits to determine the effectiveness of the kits. 

**Figure 2:**
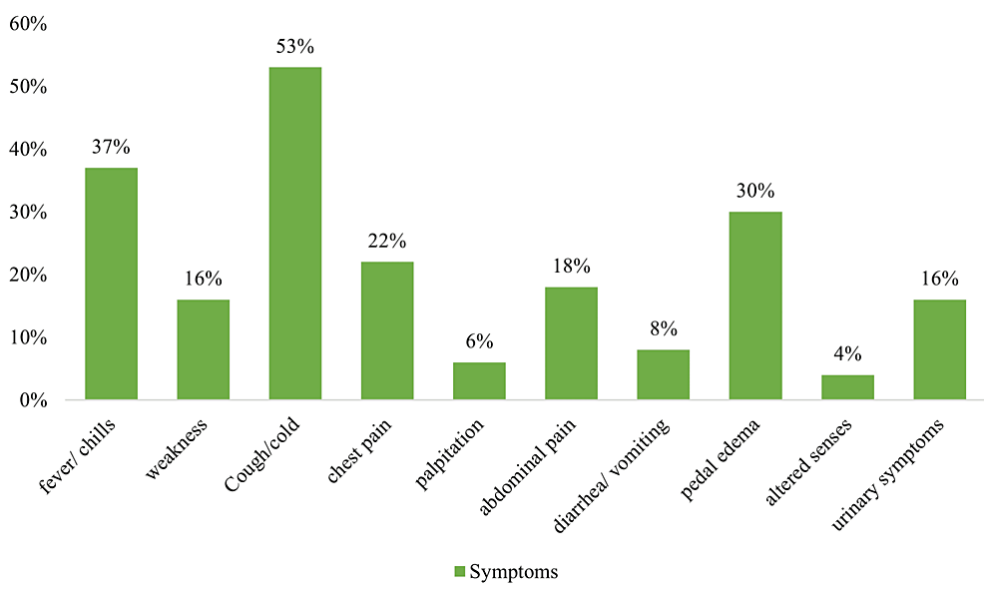
Symptoms experienced by the patients

We also investigated complaints of various symptoms among patients (Figure 2). The majority of the patients (53%; n=61) reported some physical signs and symptoms with cough or cold, 41 participants (37%) reported fever or chills, 30% (n=34) had pedal edema, 22% (n=25) experienced pain in the chest, 16% (n=18) reported weakness, and 18% (n=21) had abdominal discomfort. 

Research participants were located in hospitals and were admitted for various diseases for treatment. The majority of participants, i.e., 47.8% (n=55), were suffering from pulmonary causes, whereas 39.1% were admitted due to heart failure.

Rapid NT-ProBNP results

The participants were categorized into four variables (TP, TN, FP, and FN) by comparing the rapid kit results with the laboratory BNP levels and echocardiology findings (Table 1).

**Table 1:** NT-ProBNP results for patients presenting with dyspnea HF: Heart failure; BNP: Brain natriuretic peptide; TP: True positive; FP: False positive; FN: False negative; TN: True negative; NT-ProBNP: N-Terminal Pro-B-Type Natriuretic Peptide

Rapid test BNP levels	HF (2D Echocardiology confirmed)		Total
	Positive	Negative	
Positive	42 (TP)	47 (FP)	89
Negative	3 (FN)	23 (TN)	26
Total	45	70	115

Based on the results from Table 1, we can interpret the sensitivity, specificity, positive predictive value, and negative predictive value for the rapid kit (Table 2).

**Table 2:** Rapid test efficacy parameters Diagnostic accuracy is 37.60% (with a 95% CI of 36-39.2)

Parameter	Value	95% Confidence Interval
Sensitivity	93.30%	90.8-95.7
Specificity	32.80%	31.3-34.2
Positive Predictive Value	47.20%	45.4-47.2
Negative Predictive Value	88.40%	86-90.8

Given the high sensitivity (>90%), a negative result on this rapid test can aid in ruling out congestive heart failure as the cause of dyspnea and shifting focus towards determining other causes. However, high FP values led to a low specificity of the test. This test cannot be used to confirm heart failure as the cause of dyspnea in case of a positive result. Further investigations shall be required to establish a conclusive diagnosis.

Among the FP distribution (Table 3), the majority of the cases are due to exacerbation of chronic obstructive pulmonary disease (COPD) or asthma (none of the COPD and asthma patients gave a true negative result). These conditions have also been observed to cause an elevation of NT-proBNP levels [10-12].

**Table 3:** False positive distribution table COPD: Chronic obstructive pulmonary disease; HF: Heart failure

False Positive Etiology		N
Pulmonary	Asthma	11
	COPD	18
	pneumonia	-
	Other pulmonary causes	5
Cardiac	Cardiac causes other than HF	8
Other		5

If we test the sensitivity and specificity of elevated NT-proBNP levels (including heart failure and pulmonary causes) with the rapid kit, we can get a value of 96% and 56%, respectively, with an accuracy of 59.2% (Tables 4, 5). Hence, we can observe an increased specificity if considering only the elevated NT-proBNP levels as the primary endpoint of the rapid kit.

**Table 4:** Rapid NT-proBNP kit results compared to laboratory-determined elevated BNP levels BNP: Brain natriuretic peptide; TP: True positive; FP: False positive; FN: False negative; TN: True negative; NT-proBNP: N-Terminal Pro-B-Type Natriuretic Peptide

Rapid test BNP levels	Laboratory-tested Serum BNP levels		
	Positive	Negative	
Positive	71 (TP)	18 (FP)	89
Negative	3 (FN)	23 (TN)	26
	74	41	115

**Table 5:** Rapid test efficacy parameters considering only elevated BNP levels as the endpoint Diagnostic accuracy of 59.2% (with a 95% CI of 57.2 to 61.1) BNP: Brain natriuretic peptide

Parameter	Value	95% Confidence Interval
Sensitivity	96.00%	93.4-98.5
Specificity	56.00%	54-58
Positive Predictive Value	79.70%	77.3-82
Negative Predictive Value	88.40%	86-90.8

Age and gender distribution

**Figure 3:**
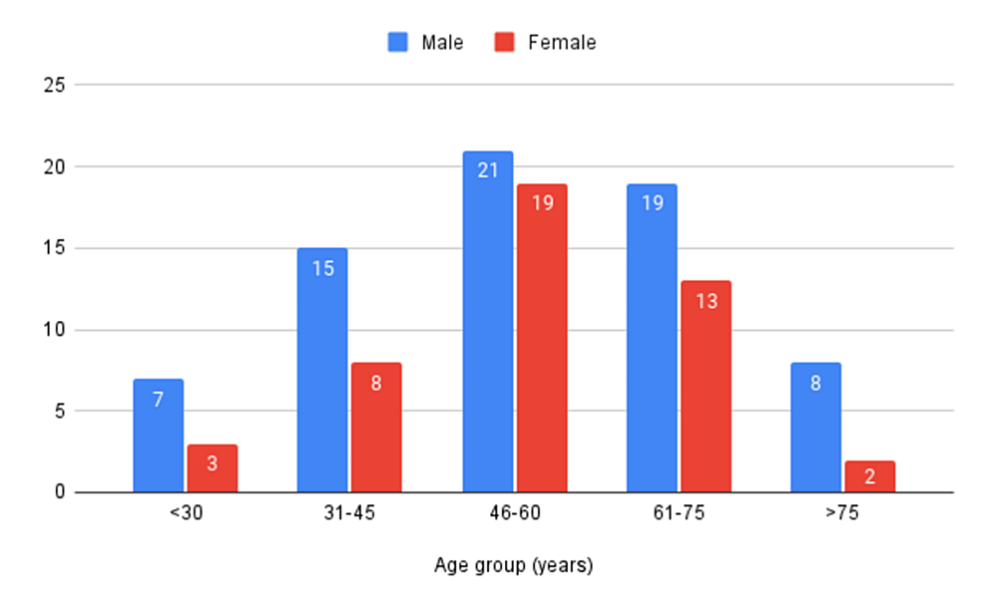
Age and gender distribution of participants

The patient sample consisted of 70 males and 45 females (Figure 3). Of these, the majority of the male population was between the age group of 46-60 years (n=21; 30%). The number of males in the age range of less than 30 years was seven (10%), with only three (6.5%) females in the same age group. Fifteen (21.5%) males were in the age range of 31-45 years, whereas females in this range were only eight (18%). The majority of females in our study belonged to the age bracket of 46-60 years (42%; n=19) and 61-75 years (29%; n=13).

The mean age of the total population was 56 years (with an SD of 16 years); the mean age of the male population was 56.3 years (with a SD of 16.8 years) and the mean age of the female population was 55.4 years (with a SD of 14.1 years). The mean difference in age between males and females is not statistically significant (p=0.76).

Dyspnea characteristics

**Figure 4:**
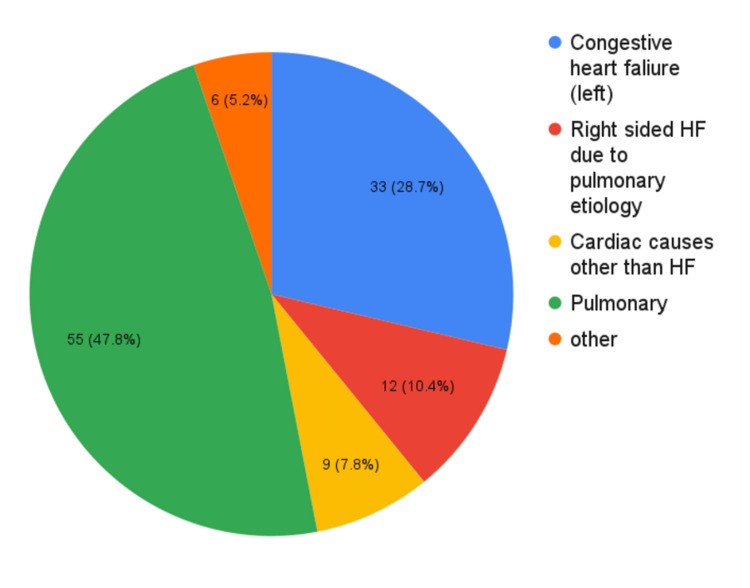
Etiologies of dyspnea presentation HF: Heart failure

Patients can present with variable characteristics of dyspnea. There are no set features of any particular disease presentation and, hence, it becomes difficult to establish a conclusive diagnosis just on the basis of dyspnea characteristics. There were some major etiologies (Figure 4) seen causing dyspnea presentation in this study.

The majority of patients presented with pulmonary causes followed by cardiac causes, and very few with other etiologies (Table 6). Pulmonary causes can be mainly divided into obstructive conditions and restrictive conditions, with the former being the majority. The most common obstructive conditions presenting with acute onset dyspnea include exacerbations of COPD and asthma. Other pulmonary conditions presenting with dyspnea include acute respiratory distress syndrome (ARDS), pulmonary artery embolism, pleural effusion, interstitial lung disease, and pneumonia. While 47.8% were suffering from pulmonary diseases, 39.1% had heart failure and 5.2% had other heart issues. Among cardiac conditions, heart failure typically presents with worsening dyspnea due to pulmonary edema over baseline shortness of breath. Ischemic coronary disease and arrhythmias can also present with shortness of breath, which is of a more sudden onset as compared to heart failure. Other causes of dyspnea include sepsis, allergies, anxiety, acidosis, etc.

**Table 6:** Distribution of etiologies of dyspnea among participants HF: Heart failure; COPD: Chronic obstructive pulmonary disease

Etiology			N	Total
					Cardiovascular	HF	Congestive HF (left)	33	45
Right-sided HF due to pulmonary etiology	12								
Cardiac causes other than HF		9	9		
Pulmonary	COPD		18	55
	Asthma		11	
	Pneumonia		12	
	Other pulmonary causes		14	
other			6	6

Distribution of dyspnea characteristics includes onset, duration, frequency, positional changes, situational changes, diurnal variations, etc. In this study, we tried to observe the duration, onset, and presence of paroxysmal nocturnal dyspnea in the patients.

Onset and duration of dyspnea

**Figure 5:**
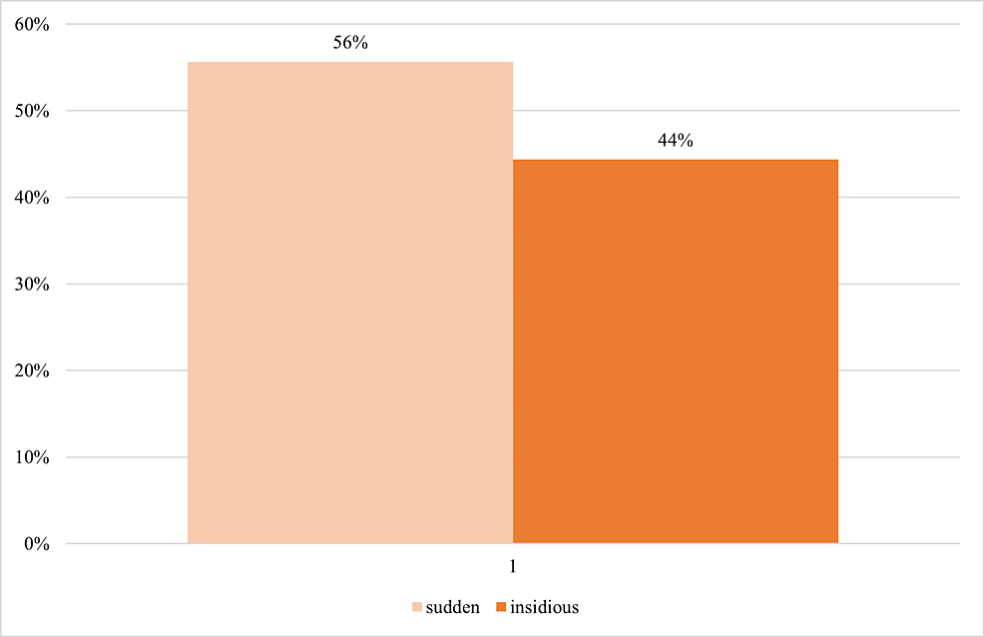
Onset of dyspnea among participants

The onset of dyspnea differs among individuals and can occur spontaneously or develop with time, which can be observed in our research participants, too (Figure 5, Table 7). The majority of participants (n=64; 56%) complained of sudden onset of the ailment without any signs while 51 (44%) participants reported gradual development of dyspnea, indicating that sudden onset of the disease is more prevalently observed. 

**Table 7:** Distribution of dyspnea characteristics with etiologies *p-value for determining difference in duration and onset for different etiologies calculated using chi-square test HF: Heart failure; PND: Paroxysmal nocturnal dyspnea

Dyspnea Characteristics			Left HF	Cardiac etiology other than HF	Pulmonary	Right HF from pulmonary etiology	Other	p-value
Duration	Acute dyspnea	>1 week	33	9	53	10	6	0.0005*
		1-7 days	16	6	20	1	3	
	Chronic	>4 weeks	-	-	4	-	-	
Onset	Sudden		22	7	30	2	3	0.004*
	Insidious		11	2	27	8	3	
PND			23	-	9	5	-	

**Figure 6:**
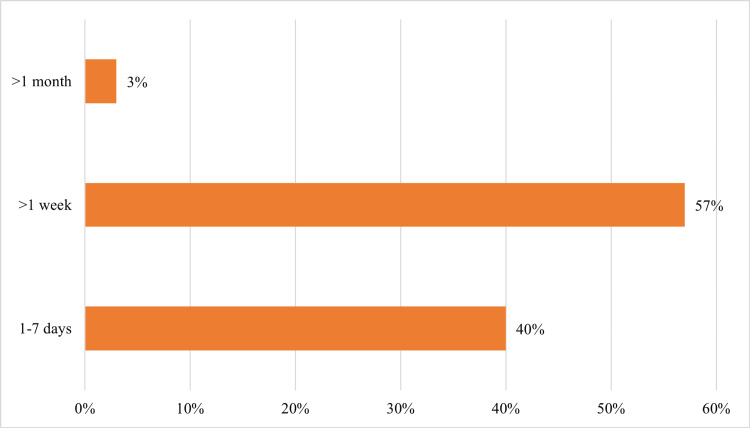
Duration of dyspnea among research participants

Forty percent (n=46) of participants presented with the complaint of experiencing dyspnea for almost the past week, 57% (n=65) of participants had shortness of breath for more than a week, whereas a minimal number (n=4) had the ailment for more than one month (Figure 6, Table 7).

Congestive heart failure is observed to have more of a sudden onset dyspnea, which is statistically significant (p=0.004). Left heart failure also has a higher percentage (69.6%) of patients with symptoms of paroxysmal nocturnal dyspnea, while this is not observed with pulmonary etiology. The majority of the cases (96.5%) presented with acute duration of dyspnea, of which a statistically significant difference was present between those presenting within a day in comparison to those presenting after one day (p=0.0005).

Association with NYHA class

We tried to describe the severity of dyspnea, including the presence of dyspnea with exertion. The higher the NYHA class, the more severe the dyspnea presentation in the patient. Heart failure patients typically presented with greater severity of dyspnea, indicated by the majority of cases presenting with class 3 and class 4 NYHA (Table 8).

**Table 8:** Classification of NYHA and correlation with BNP kit levels *p-value for determining difference between increasing severity of dyspnea (increase NYHA class) and increasing BNP levels is calculated using chi-square test NYHA: New York Heart Association; BNP: Brain natriuretic peptide

NYHA class	BNP levels (pg/ml)				Total	p-value
	>450	>1500	>2500	>5000		
1	-	-	-	-	-	0.006
2	1	1	1	0	3	
3	0	1	2	14	17	
4	0	2	2	16	20	
Total	1	4	5	30	40	

**Figure 7:**
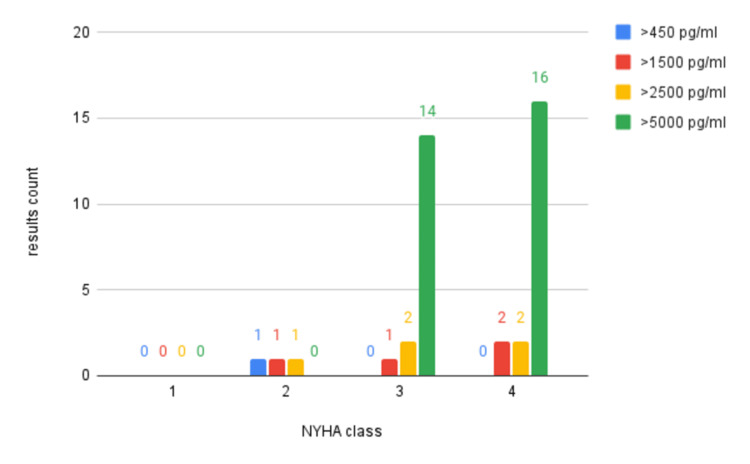
Comparison of severity of NYHA with increasing NT-ProBNP levels NYHA: New York Heart Association; NT-ProBNP: N-Terminal Pro-B-Type Natriuretic Peptide

Of the total TPs (40 patients with heart failure who tested positive on the rapid kit), it was observed that the BNP levels increased with increasing severity of dyspnea (i.e., increasing NYHA class), and the findings were statistically significant (p<0.05) (Table 9, Figure 7). This shows that the rapid kit can also aid in determining the severity of heart failure based on the severity of dyspnea in patients presenting in the emergency department.

**Table 9:** NYHA and HF classification among participants NYHA: New York Heart Association; HF: Heart failure

NYHA	Congestive HF	Right HF
class 1	-	-
class 2	6	1
class 3	12	4
class 4	14	5

Association with co-morbidities 

There was no statistically significant difference observed between the severity of dyspnea and the presence of co-morbidities in heart failure patients (p>0.05). The presence of co-morbidities in heart failure patients was not found to be associated with increased severity of dyspnea (Table 10).

**Table 10:** Comorbidities reported among research participants *p-value for difference between dyspnea severity and association with co-morbidities was determined using chi-square test NYHA: New York Heart Association; HTN: Hypertension; DM: Diabetes mellitus

NYHA	HTN	DM	Total	p-value
1	-	-		0.49*
2	2	0		
3	9	4		
4	7	5		
Total	18	9	27	

Blood pressure was measured in the participants during their hospital visit, which was kept on record for the study(Table 11). The data elucidated that 46% of participants had low blood pressure, indicating that dyspnea and shortness of breath might cause decreased blood pressure or vice versa.

**Table 11:** Blood pressure of patients

Blood Pressure Range Systolic/ Diastolic mmHg	Interpretation	N	%
<120/ <80	Decreased	53	46%
120/80	Normal	20	17%
120-139/ 80-89	Hypertension Stage-1	12	10%
140-180/ 90-120	Hypertension Stage- 2	25	22%
>180/ >120	Hypertension Crisis	5	4%

Low blood pressure in patients might act as a contributory factor to dyspnea with decreased circulation of blood. The correlation coefficient was -0.7, indicating that low blood pressure can potentially cause the increased presence of dyspnea, but the p-value was 0.1 (>0.05), indicating that this was not statistically significant. Among other participants, 22% (n=25) experienced elevated blood pressure, and 4% (n=5) were admitted due to their hypertension crisis.

The majority of patients had normal pulse rates, whereas 34% (n=39) participants had an increased pulse rate as compared to others. Their heart rate was recorded to be elevated to 100 beats per minute, which is more than the normal range of 60-100 beats per minute by the British Heart Foundation [13].

The saturation of oxygen (SpO2) in blood was also evaluated during the hospital visit, which was kept on record for analysis. Four percent reported very low SpO2, less than 50% (n=3); 17% (n=19) reported SpO2 in the range of 51-80, and 34% (n=39) had SpO2 in the range of 81-90. The SpO2 among the majority of participants, 47% (n=54), was above 90. The normal range of SpO2 is between 94-98% in patients, according to the British Thoracic Society [14].

## Discussion

Making the correct diagnosis in suspected heart failure patients is crucial but also challenging. Studies have shown that in addition to routine history, clinical examination, and routine investigations such as chest radiography and echocardiography, measurement of natriuretic peptide levels improves diagnostic accuracy in differentiating the various causes of acute dyspnea in an emergency setting [15]. Brain Natriuretic Peptide (BNP) is a physiologic marker that is secreted by the ventricular cardiac myocytes in response to stretch that occurs due to ventricular distension and overloading. Myocytes release pre-ProBNP, which is cleaved into BNP and NT-proBNP, which opposes the actions of the renin-angiotensin-aldosterone system (RAAS) and causes diuresis, natriuresis, and vasodilation. BNP or NT-proBNP levels can be measured with a blood laboratory assay test, and studies have demonstrated that elevated levels of BNP (> 100/ml) or NT-proBNP (>300 pg/ml) are associated with major adverse cardiac events [16]. In fact, the increasing proBNP levels are also associated with the increasing severity of dyspnea, which was observed in this study. However, in a patient presenting with dyspnea, possibly due to acute heart failure, the diagnosis can be delayed due to untimely serum BNP results or the unavailability of 2D echocardiography in the emergency department. Lately, there have been developments with the rapid BNP testing kits to overcome these issues [17], and the results from our study highlight its effectiveness in ruling out heart failure as a cause of dyspnea, which can aid in redirecting resources toward establishing other conclusive diagnoses.

Congestive heart failure symptoms can be perceived with variable intensity in different clinical settings, and therefore, it becomes important to assess the severity of CHF using BNP levels in accordance with the NYHA class. BNP levels of >100 pg/ml are considered abnormal, and levels >5000 pg/ml are associated with poor prognosis [18]. The rapid kit used in this study also helped in differentiating the severity of heart failure and the respective BNP levels based on the obtained color gradient. This aids in diverting immediate attention towards patients with poorer conditions and subsequent timely management. The characteristics of dyspnea can vary due to different underlying disease processes. Based on the duration, it is mostly divided into acute, which is present for less than four weeks, and chronic, which is for more than four weeks. The majority of the cases presenting in an emergency setting tend to be acute [19], which was also observed in this study.

Patients can present with an insidious onset of dyspnea or can have a sudden exacerbation of existing chronic shortness of breath. They can also have episodic attacks and changes in dyspnea with positional differences. Underlying pulmonary infection can also make it tough to clinically differentiate it from heart failure [20]. Because of these variable presentations, it becomes difficult to establish the cause of dyspnea just on the basis of the dyspnea characteristics, physical findings, and history [21]. The presence of co-morbidities is also associated with increasing severity of heart failure presentation, of which the most important ones are hypertension and diabetes mellitus type 2 [22]. Diabetes is also a major factor in determining the prognosis of heart failure patients. Aggressive glycemic control has been shown to prevent worsening outcomes [23]. This study was, however unable to determine any significant correlation between the presence of hypertension and diabetes with increased severity of heart failure.

Shortness of breath can mainly be due to pulmonary pathology or cardiovascular conditions, and it is imperative to differentiate between these due to different management approaches [19]. Acute dyspnea is often misdiagnosed initially, which leads to a longer duration of hospitalization and increased mortality [24]. One of the methods to differentiate cardiac and pulmonary causes of shortness of breath is to use the Dyspnea Discrimination Index (DDI) in the emergency department. DDI is the product of peak expiratory flow rate and oxygen partial pressure divided by 1000. Chandy et al. effectively showed that DDI is lower in pulmonary causes of dyspnea than in cardiac etiology [25]. It is observed that pulmonary causes of dyspnea have better outcomes than cardiac causes as the former can be stabilized by initial emergency management with oxygen and bronchodilators, but cardiac conditions have a more acute presentation needing further workup and interventions [26], which was also reported in this study. Of the cardiac causes, the most common ones to present with acute dyspnea include heart failure, acute coronary ischemic events, and arrhythmias. Ischemias and arrhythmias can be picked up on an ECG during the initial evaluation, but heart failure diagnosis needs serum elevated BNP levels or an echocardiogram to determine the low ejection fraction.

The rapid test used in this study had a high sensitivity, which can potentially aid in ruling out heart failure as a cause of dyspnea if the results are negative, forgoing the time-consuming evaluation with an echocardiogram [27]. Specificity was low due to higher FP results, which were mainly due to pulmonary cause. Studies have shown that acute exacerbations of COPD and asthma can lead to a mildly transient elevation of NT-proBNP levels [10-12]. This explains the higher incidence of FPs in this study pertaining to an equally high number of patients presenting with dyspnea due to pulmonary etiology. Hence, it can be proposed that even though these tests have a low specificity for establishing heart failure as the conclusive cause of dyspnea, they do have a high specificity when it comes to detecting elevated NT-proBNP levels in the blood.

SpO2 is measured primarily by pulse oximeters easily accessible at healthcare facilities. Smith et al. conducted a study based on measurements in 37,593 acute medical admissions concerning air inhalation and suggested a higher SpO2 target of 96-98% as normal oxygen saturation, and indicated that SpO2 below 90 required immediate attention and medical care to avoid hypoxia-induced cell death in human body tissues [28]. We had 3% patients with less than 50 and 17% patients with less than 80 as well as 34% with SpO2 of 81-90, demonstrating low SpO2 among patients that can cause chronic hypercapnia and hypoxemia, which might act as contributing factors for severe consequences of oxygen deprivation in cells. It can be said that lack of SpO2 in blood is associated with the decreased functioning of the heart due to disease as well as other causes [29]. Another study by Van Riet et al. determined the association of shortness of breath with increased pulse rate and NT-proBNP. It included patients of more than 65 years with 12 months of follow-up. The study elucidated that NT-proBNP levels were elevated for the advent of heart failure as well as incidence and identification of heart failure, which was 2.9% among participants, indicating exertion and shortness of breath are indicators of unmarked heart failure [30]. Similarities were observed in our evaluation of increased blood pressure and increased pulse as well as decreased SpO2 among participants.

Early measurement of BNP levels in patients presenting with dyspnea is also shown to reduce the total expenses of patient care for both the patient as well as the hospital [31]. More studies on the rapid test kits are essential in different groups of the population and with an increased sample size to achieve even better efficacy than what was observed in our study. This test cannot replace 2D echocardiography for determining the ejection fraction, but it can definitely aid in prompt action and better outcomes, especially in healthcare settings with a lack of necessary equipment. These kits can be used in rural settings where patients can be managed more optimally in spite of a lack of infrastructure and basic facilities. Availability of these in peripheral areas can help establish baseline etiologies and, in turn, provision of stability measures till the patient is referred to higher centers. Further studies in this field of rapid diagnostics would also support the rationale behind mass production and, in turn, more economical advantage for patients as well as healthcare setups.

## Conclusions

Patients presenting with acute dyspnea can mainly be due to pulmonary or cardiac cause and the latter are more threatening due to their differences in management outcomes. One of the major causes of cardiac dyspnea is congestive heart failure, which can be determined by measuring serum BNP or NT-proBNP levels. Higher levels often indicate increased severity of the condition and a poorer prognosis. Lately, rapid diagnostic kits are being studied to determine the presence and the level of NT-proBNP levels in the emergency department for patients presenting with dyspnea potentially due to heart failure, and these can be useful for narrowing down the diagnosis of dyspnea. This study showed a high sensitivity of the rapid testing kits (>90%), which can aid in ruling out heart failure in case of negative results. This study also showed that BNP levels can be determined using these kits, which can in turn, indicate the severity of heart failure and symptoms in general. It was observed that increased severity of congestive heart failure is associated with increased BNP levels. Overall, these kits can be an effective and economical tool to narrow down the differential diagnosis of dyspnea, especially in resource-poor healthcare setups.
